# Supplementary material for: Diagnosis and management of jejunoileal diverticular haemorrhage: An update on the experience in a single centre
Source: PLoS One. 2020 Jun 23;15(6):e0234417. doi: 10.1371/journal.pone.0234417 (PMC7310693; doi:10.1371/journal.pone.0234417)
Supplement: Attachment — Submitted filename: Response to Reviewer.docx [file pone.0234417.s002.docx]

Chun Chieh Yeh, M.D., Ph.D.

Academic Editor

PLOS ONE

Dear Editor,

We appreciate your editorial comments, as well as those of the reviewers, concerning our manuscript. Based on these comments, we have made several revisions to our manuscript, which is resubmitted for your consideration. The manuscript has also been revised according to the PLOS ONE's style requirements. If there is anything needing to be further improved, please do not hesitate to inform us at your earliest convenience. Your assistance is highly appreciated. We look forward to your message.

The followings are point-by-point responses to the comments.

Journal requirements:

When submitting your revision, we need you to address these additional requirements.

1. Please ensure that your manuscript meets PLOS ONE's style requirements, including those for file naming. The PLOS ONE style templates can be found at

https://journals.plos.org/plosone/s/file?id=wjVg/PLOSOne_formatting_sample_main_body.pdf and https://journals.plos.org/plosone/s/file?id=ba62/PLOSOne_formatting_sample_title_authors_affiliations.pdf.

**Response:** The manuscript has been revised per the journal’s requirement. If there is anything further required, please do not hesitate to inform us. We are very happy to cooperate.

2. We note that you have included the phrase “data not shown” in your manuscript. Unfortunately, this does not meet our data sharing requirements. PLOS does not permit references to inaccessible data. We require that authors provide all relevant data within the paper, Supporting Information files, or in an acceptable, public repository. Please add a citation to support this phrase or upload the data that corresponds with these findings to a stable repository (such as Figshare or Dryad) and provide and URLs, DOIs, or accession numbers that may be used to access these data. Or, if the data are not a core part of the research being presented in your study, we ask that you remove the phrase that refers to these data.

**Response:** We understand the policy of the journal. All authors agreed to provide de-identified raw data as supporting information from which all of the results were derived. The phrase “data not shown” has been removed.

Reviewers' comments:

Reviewer's Responses to Questions

Comments to the Author

1. Is the manuscript technically sound, and do the data support the conclusions?

The manuscript must describe a technically sound piece of scientific research with data that supports the conclusions. Experiments must have been conducted rigorously, with appropriate controls, replication, and sample sizes. The conclusions must be drawn appropriately based on the data presented.

Reviewer #1: Yes

Reviewer #2: Partly

2. Has the statistical analysis been performed appropriately and rigorously?

Reviewer #1: Yes

Reviewer #2: Yes

3. Have the authors made all data underlying the findings in their manuscript fully available?

The PLOS Data policy requires authors to make all data underlying the findings described in their manuscript fully available without restriction, with rare exception (please refer to the Data Availability Statement in the manuscript PDF file). The data should be provided as part of the manuscript or its supporting information, or deposited to a public repository. For example, in addition to summary statistics, the data points behind means, medians and variance measures should be available. If there are restrictions on publicly sharing data—e.g. participant privacy or use of data from a third party—those must be specified.

Reviewer #1: Yes

Reviewer #2: No

**Response:** All authors agreed to provide de-identified raw data as supporting information from which all of the results were derived.

4. Is the manuscript presented in an intelligible fashion and written in standard English?

PLOS ONE does not copyedit accepted manuscripts, so the language in submitted articles must be clear, correct, and unambiguous. Any typographical or grammatical errors should be corrected at revision, so please note any specific errors here.

Reviewer #1: Yes

Reviewer #2: Yes

5. Review Comments to the Author

Reviewer #1:

To the authors,

This manuscript is a very interesting and valuable study in the GI field. As we know, the jejunoileal diverticular bleeding is not common in the clinical practice, especially in the Western countries. By the contrast, the jejunoileal diverticular bleeding is more common in the Asian countries compared to the Western countries.

I have some questions for your manuscript

Q1. In the section: Enteroscopy is the primary diagnostic approach for jejunoileal diverticular haemorrhage (JIDH) and includes endoscopy of the small intestines [6], small bowel follow-through studies, computed tomography (CT), angiography and technetium red cell-tagged scans that are beyond the reach of esophagogastroduodenoscopy and colonoscopy [1]. Its meaning is not very clear to readers. Can you rewrite them ?

**Response:** All authors thank the reviewer’s correction. We apologize for the misleading and somewhat mistaken sentence. The sentence has been rewritten as follows, “*Enteroscopy is the current primary endoscopic diagnostic and therapeutic approach for obscure gastrointestinal bleeding [6] including jejunoileal diverticular haemorrhage (JIDH). Alternative non-endoscopic diagnostic tools, including small bowel follow-through studies, computed tomography (CT), angiography and technetium red cell-tagged scans, are used when enteroscopy is not available [1]*,” as in line 49-53.

Q2. Your manuscript mentioned "Investigation revealed coexisting gastrointestinal diverticula in the duodenum (23.53%) and colon (14.71%)". Can I ask the diagnostic tool for your coexisting gastrointestinal diverticula?

**Response:** We thank the reviewer for the question. Because of higher incidence as well as anatomical accessibility of the gastro-duodenal and colonic hemorrhage, patients with gastrointestinal bleeding will received either esophagogastroduodenoscopy or colonoscopy before further investigation for small intestinal bleeder. Hence, those duodenal and colonic diverticula were found by esophagogastroduodenoscopy or colonoscopy. Please find line 116 where we have added a sentence to address this concern.

Q3. In your manuscript, you said that deep enteroscopy was introduced at your institution in 2004 for the treatment of small intestinal haemorrhage. What is your reason for the decreased rate in diagnostic enteroscopy after 2010? Moreover, what is your diagnostic enteroscopy before 2004?

**Response:** The authors thank the reviewer for the question. The management of obscure GI bleeding changed over the past two decades. For example, in the AGA medical position statement (Gastroenterology. 2000 Jan;118(1):197-201. DOI: 10.1016/s0016-5085(00)70429-x), there was no role of CT scan in diagnosis/management for OGIB.

The situation changed after the introduction of deep enteroscopy. In fact, deep enteroscopy was introduced to Taiwan and our institution in 2004. Before 2004, we performed push endoscopy which could only visualize the proximal jejunum at most. As the resolution and feasibility of computed tomography angiography (CTA) improved (Yen HH et al. World J Gastroenterol. 2012 Feb 21;18(7):692-7. doi: 10.3748/wjg.v18.i7.692), patients usually received non-invasive diagnostic procedure such as CTA first, followed by invasive diagnostic procedures such as enteroscopy.

Q4. In the diagnostic modalities of jejunoileal diverticula, your described the total number was 64 before 2009, and 60 after 2010. Does the number mean the patients numbers? (your total enrolled patients were 68)

**Response:** All authors thank the reviewer for the correction. The number “64” and “60” were the sums of the counts of the diagnostic procedures. They were not referred to as the number of patients, and they were therefore negligible. We would like to remove the row of the table in order not to cause confusion as well as unnecessity. The total numbers of patients before 2009 and after 2010 were 35 and 33, respectively, which we would like to add in line 153 and 156.

Reviewer #2:

This is a retrospective study about jejunoileal diveticular hemorhage. the study included small number of patients- 68 only.

some major revisions need to be done:

1- the abstract should be fragmented into introduction, methods, results and conclusion- in breif as the whole article.

**Response:** The authors thank the reviewer for the correction. The abstract has been rewritten as a structured abstract.

2- in the introdcution section: enteroscopy is an endoscopy of the small bowel and does not include small bowel follow through, CT scan, angiography and Tc RBC scan- these are a different radiological dignostic tools, and not as written that are part of the enteroscopy.

**Response:** All authors thank the reviewer’s correction. We apologize for the misleading and somewhat mistaken sentence. The sentence has been rewritten as follows, “*Enteroscopy is the current primary endoscopic diagnostic and therapeutic approach for obscure gastrointestinal bleeding [6] including jejunoileal diverticular haemorrhage (JIDH). Alternative non-endoscopic diagnostic tools, including small bowel follow-through studies, computed tomography (CT), angiography and technetium red cell-tagged scans, are used when enteroscopy is not available [1]*,” as in line 49-53.

3- following the introduction, methods and materials should be written before the results.

**Response:** The authors thank the reviewer for the correction. The “materials and methods” section has been shifted right after the “introduction” section.

4- the most utilised diagnostic procedure was CT scan according to the authors- the authors means CT scan or CT angiography scan? and wether CT angiography was used as a diagnostic procedure.

**Response:** All authors thank the reviewer for the question. In our institution, abdominal CT scan indicated for GI bleeding is conducted with CT angiography protocol (Yen HH et al. World J Gastroenterol. 2012 Feb 21;18(7):692-7. DOI: 10.3748/wjg.v18.i7.692), because non-contrast CT scan is not helpful. Therefore, in the present study, all CT scans were performed as CT angiography. We did not clarify this point in the manuscript and we believe the same question would be raised by readers. We would like to add a sentence, “*All of the CT scans in the present study were performed with CT angiography protocol*”, in line 89 and 90, to avoid such concern.

5- what were the findings on the different diagnostic tools?

**Response:** Different diagnostic tools in the present study included enteroscopy, CT scan (CT angiography), angiography, surgery, and small bowel flow-through study. By enteroscopy, bleeders or lesions could be directly visualized with in small intestines. CTA scan is able to diagnose and localize the nature of the bleeder, such as tumor or diverticulum. Angiography is used to localize the bleeder with contrast extravasation, yet with inferior diagnostic capability to outline non-vascular lesions or structures. Exploratory surgery was commonly conducted under emergency and could directly expose the lesions followed by surgical repair. Finally, small bowel flow-through study utilizes contrast medium to delineate the intestinal lumen, and therefore could facilitate diagnosis of bowel structural abnormality such as diverticulum, unable to confirm the presence of bleeding or the nature of the bleeder.

6- 22 patiens were treated initially by surgical means- but afterward, surgery was used in total of 7 patients for diagnosis... this means that 15 patients actually underwent other dianostic procedures- what are these procedures? and what was the indication for operation?

**Response:** The authors thank the reviewer’s comment. Overall, 22 patients were treated with surgery, and with all due respect, diagnostic surgery had been performed in 2, but not 7, patients. In the other 20 patients, these diagnostic procedures included CT scan in 13 patients, angiography in 5 patients, and endoscopy in 2 patients. The indications for surgery were jejunoileal diverticular bleeding based on individualized clinical situations.

7- table 3 should include the total number of patients before 2009 and total number after.

**Response:** The authors thank the reviewer’s suggestion. The total numbers of patients before 2009 and after 2010 were 35 and 33, respectively. However, a patient could have undergone more than one diagnostic procedures. We are afraid that if the total number of patient be written in Table 3, the readers might be confused with the total number of the patients or the procedures. Therefore, please allow us to remove the total number from Table 3. Alternatively, we would like to add the total number of patients in the text, as in line 153 and 156.

Thank you for the opportunity to resubmit this manuscript for consideration of publication in PLOS ONE. If you have any questions or comments regarding this manuscript, please do not hesitate to contact us by mail at our correspondence address, by fax at +886-4-7228289, by telephone at +886-4-7238595ext5501, or by e-mail at 91646@cch.org.tw

Sincerely,

Hsu-Heng Yen M.D

Department of Gastroenterology

Changhua Christian Hospital, Taiwan.
